# Emotional arousal modulates oscillatory correlates of targeted memory reactivation during NREM, but not REM sleep

**DOI:** 10.1038/srep39229

**Published:** 2016-12-16

**Authors:** Mick Lehmann, Thomas Schreiner, Erich Seifritz, Björn Rasch

**Affiliations:** 1 Department of Psychiatry, Psychotherapy and Psychosomatics, Psychiatric Hospital University of Zurich, Switzerland; 2 Institute of Psychology, University of Zurich, Zurich, Switzerland; 3 Zurich Center for Interdisciplinary Sleep Research (ZiS), University of Zurich, Switzerland; 4 Radboud University, Donders Institute for Brain, Cognition and Behaviour, Nijmegen, The Netherlands; 5 Department of Psychology, University of Fribourg, Fribourg, Switzerland

## Abstract

Rapid eye movement (REM) sleep is considered to preferentially reprocess emotionally arousing memories. We tested this hypothesis by cueing emotional vs. neutral memories during REM and NREM sleep and wakefulness by presenting associated verbal memory cues after learning. Here we show that cueing during NREM sleep significantly improved memory for emotional pictures, while no cueing benefit was observed during REM sleep. On the oscillatory level, successful memory cueing during NREM sleep resulted in significant increases in theta and spindle oscillations with stronger responses for emotional than neutral memories. In contrast during REM sleep, solely cueing of neutral (but not emotional) memories was associated with increases in theta activity. Our results do not support a preferential role of REM sleep for emotional memories, but rather suggest that emotional arousal modulates memory replay and consolidation processes and their oscillatory correlates during NREM sleep.

Emotional events are highly relevant for adaptive behavior and therefore better encoded and remembered than neutral ones^1^. According to the widely accepted memory modulation hypothesis^2^, activation of the amygdala by emotions during encoding modulates plastic processes in memory-related brain regions including the hippocampus, thereby increasing subsequent consolidation processes. In support of this notion, emotionally arousing learning material is better remembered than neutral stimuli^3^, and this emotional memory advantage increases over time^4^,^5^, suggesting a continuing influence of emotional arousal on ongoing consolidation processes.

In addition to emotional arousal, sleep has also been critically implicated in the consolidation of memories. The beneficial effect of sleep on memory occurs presumably due to a spontaneous reactivation of newly encoded memories during sleep^6^. According to the active system consolidation hypothesis^7^, hippocampal memory reactivations during Non-rapid eye movement (NREM) sleep support the strengthening and integration of memories into cortical long-term stores. Several authors have suggested that this replay process during sleep is selective, thereby specifically strengthening those memories that are relevant for our future behavior^8^,^9^. Supporting this concept, sleep selectively strengthens those memories associated with reward or expected to be of future relevance^10^,^11^. Importantly, several studies have reported a preferential or even selective benefit of sleep for emotional memories^12^. And while rewarded memories seem to be preferentially replayed during NREM sleep^13^, direct evidence for the preferential replay of emotional memories during sleep in humans is still lacking.

The stage of REM sleep has been long considered of high importance for the consolidation of emotional memories. Dream reports from REM sleep are often vivid and highly emotional^14^ and their content is potentially involved in memory reprocessing^15^,^16^,^17^. Moreover, several empirical studies have provided evidence for a critical role of REM sleep for emotional memories: A growing body of research in rodents consistently shows that REM sleep increases after an emotional learning task (e.g. fear conditioning^18^). In some cases, however, fear conditioning produced a selective suppression of subsequent REM sleep^19^,^20^. In humans, studies using the night-half paradigm show that emotional memories are particularly strengthened after late-REM sleep rich sleep^21^,^22^,^23^,^24^ and higher amounts of REM sleep predict increased emotion memory performance^25^ and their neural correlates^26^,^27^. On a theoretical level, it has been proposed that REM sleep represents a neurobiological brain-state particularly beneficial to selective emotional memory processing^28^,^29^,^30^: For example, limbic regions including the amygdala show increased activity during REM sleep^31^,^32^, which might reflect a reactivation of emotionally salient memories during REM sleep^33^. Furthermore, increased cholingeric activity during REM sleep might enhance amygdala-dependent memory reprocessing^30^. In addition, characteristic theta oscillations during REM sleep have been linked to the consolidation of emotional memories^34^. Theta rhythm might coordinate the replay of emotional memories in different brain regions that were initially involved in encoding various aspects of emotional experiences^25^,^35^ and promote their integration into pre-existing autobiographical memory networks^36^,^37^,^38^. Finally, the entire process of emotional reprocessing during REM sleep might possibly be related to dreaming activity during this sleep stage^30^. In spite of this theoretical assumption, a causal role of memory reactivations during REM sleep for emotional memory consolidation has not yet been shown. Replay processes during REM sleep have been observed in rodents^39^ but it remains unclear whether they serve the same function as during NREM sleep^40^ or if they are exclusively beneficial for emotional memories.

An established technique to examine this questions is targeted memory reactivation (TMR) during sleep, which consists of inducing reactivation mechanisms by presenting memory cues during sleep^41^. Using TMR, it has been consistently shown that re-exposure to memory cues during NREM (but not during REM) sleep improves neutral declarative memory consolidation^42^,^43^,^44^,^45^,^46^,^47^. In addition, we could show by using verbal memory cues that the strengthening of memories by cueing during NREM sleep is associated with an increase in theta as well as spindle activity^45^,^48^. Studies using TMR for emotional memories are scarce and reveal quite inconsistent results. In rodents, repeated presentation of conditioned cues during sleep increases fear memories during both REM^49^ and NREM sleep^50^,^51^,^52^. In humans, reactivating fear condition memories during NREM reduced memory for fear^53^, while inducing reactivations of fear memories or emotional faces during REM sleep had no effect^46^,^54^,^55^. A possible reason for inconsistent results might be that no study so far systematically compared reactivation success for emotional memories during (deep) NREM and REM sleep. In addition, the oscillatory correlates of successful emotional memory reactivation during sleep are still completely unclear.

Here we systematically tested the effect of cueing memories during REM and NREM sleep as well as wakefulness on emotional memory formation. We hypothesized that TMR of emotionally arousing memories during REM sleep selectively improves emotional memories associated with increases in oscillatory theta activity. In addition, the benefits of cueing emotional memories during REM sleep should exceed the cueing benefits obtained during NREM sleep. In contrast to our hypotheses, our results show that TMR robustly induced a preferential cueing benefit for emotionally arousing memories during NREM sleep, which was associated with increased theta and spindle activity and more often followed by slow waves. During REM sleep, we observed no significant cueing benefit for emotional memories, and an increase in theta for successfully cued neutral but not emotional memories. No increases were observed for spindle oscillations during REM sleep. Our results do not support a specific role for reactivations during REM sleep for emotional memory consolidation, but rather suggest that emotional arousal modulates reactivation processes underlying declarative memory consolidation during NREM sleep.

## Results

**Figure 1:**
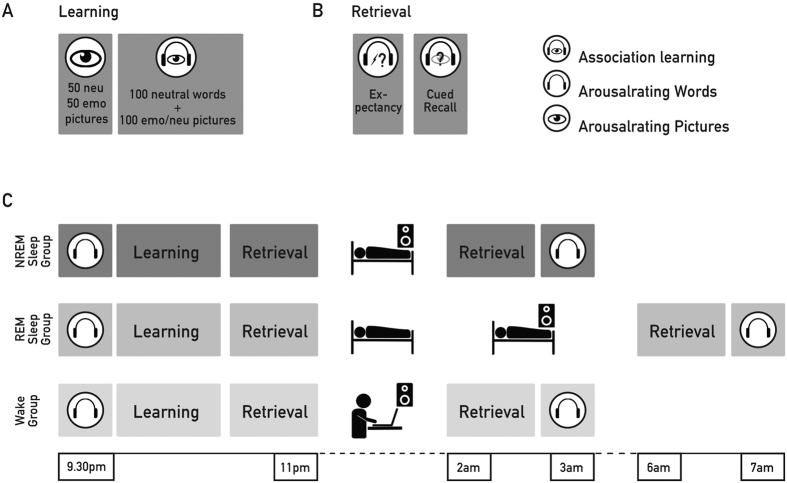
Experimental Procedure. (**A)** Before learning, all participants first rated the arousal of 100 neutral words and 50 arousing (emotional, emo) and 50 non-arousing pictures (neutral, neu), respectively. Then they performed on the emotionally association learning task. In this task, first a neutral word was presented via loudspeakers, followed by the presentation of an emotionally arousing or neutral picture. The same word was presented a second time during the picture presentation. Participants performed on three rounds: No response was required in the first round. In the second and third round, participants rated the expectancy of an arousing pictures after the first presentation of the word. In any case, following the rating, the correct picture was presented again serving as feedback. (**B)** During retrieval, participants heard the word and had to indicate the expectancy of an arousing picture (Expectancy) and provide a brief written description of the picture (Cued Recall). (**C)** Retrieval performance was assessed immediately after learning and after the retention interval. During the 3-h (NREM Sleep and Wake Group) or 6-h retention interval (REM Sleep Group), single word cues were presented repeatedly for 80 minutes via loudspeakers, either during NREM, REM or during performance of a computer working memory task. At the end, subjects rated the arousal level of words again.

We tested our hypotheses using a newly developed emotional associative learning task: Sixty-two healthy young participants learned to associate spoken neutral words with a picture shown on a computer screen. The learning material for each subject consisted of 100 neutral words of which 50 words were associated with emotionally arousing pictures and the remaining 50 words were associated with neutral pictures. From each category, emotional and neutral, half of the words were randomly and repeatedly presented either during NREM sleep (*n* = 21), REM sleep (*n* = 20) or wakefulness (*n* = 21), in a between-subject design. Cued recall was tested after the retention interval, during which each word was presented auditory and participants were asked to write down a short description of the associated picture (see Fig. 1, for a summary of the experimental procedure and design).

### Behavioral performance

#### Retention performance and cueing benefits

**Figure 2:**
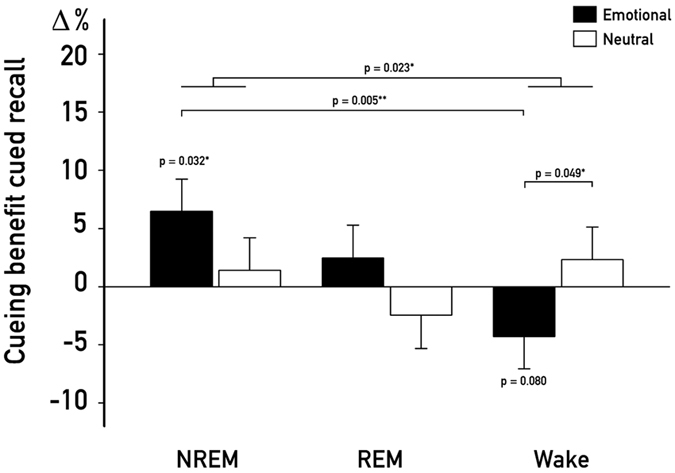
Cued Recall. Differences in cueing benefits for emotional and neutral associations. Cueing of emotional associations (black bars) improved memory recall only when applied during NREM (P = 0.032), but not during REM sleep or wakefulness. Cueing benefits for emotional associations were greater for the NREM as compared to the Wake group (P = 0.005). Independent of arousal, cueing benefits were generally greater for the NREM as compared to the Wake group (main effect of cueing: P = 0.023), while no general cueing benefit emerged for REM sleep (P = 0.84). The cueing benefit score was created by first calculating the change of correctly recalled associations for cued and uncued associations separately, with setting the performance before the retention interval to 100%. Following, the uncued was subtracted from the cued score. Values are means ± SEM. *P ≤ 0.05 **P ≤ 0.01.

To test our main hypothesis, we analyzed the benefits of cueing during different brain states (NREM, REM and wake) on memory for emotionally arousing and neutral associations using a 3 × 2 analysis of variance. As dependent variable we used the cueing benefit score (correctly recalled cued minus uncued associations, with learning performance set to 100%). As expected, only in the NREM sleep group, cueing benefits were significantly different from zero, i.e. cued associations were better remembered than uncued associations (NREM: *P* = 0.032, REM: *P* = 0.982; Wake: *P* = 0.692, see Fig. 2). Moreover, we observed a significant interaction between “group” and “emotional valence” (*F*_*1,59*_ = 3.17, *P* = 0.049, 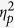 = 0.102), which was mainly driven by a significantly greater cueing benefit for emotionally arousing associations after cueing during NREM sleep as compared to wakefulness (planned post-hoc pairwise comparison for emotional associations: NREM vs. wake: *t*_*1,40*_ = 2.95, *P* = 0.005, 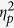 = 0.17, REM vs. wake: *t*_*1,39*_ = 1.75, *P* = 0.088, 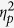 = 0.07; NREM vs. REM: *t*_*1,39*_ = 0.96, *P* = 0.343, 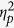 = 0.02, see Fig. 2). However, cueing of neutral associations did not benefit memory performance in any group (all *P* ≥ 0.26). Memory performance for uncued associations did not differ between the NREM and the REM sleep group (*t*_*1,39*_ = 1.18, *P* = 0.244), indicating that the longer sleep duration in the REM sleep group (see Table 1) did not influence general memory consolidation of associated pictures in our task.

Descriptively in the wake group, cueing of emotionally arousing associations impaired memory performance, while this “negative cueing benefit” did not reach significance (*P* = 0.080). For neutral associations, no significant effects emerged (all *P* ≥ 0.20).

As opposed to cued recall performance, expectancy ratings for emotional and neutral associations were not affected by cueing, irrespective whether it occurred during NREM, REM or wakefulness (*F*_*2,59*_ = 0.50, *P* = 0.61). Analyzing the three groups separately revealed no sleep stage specific cueing, emotional arousal or interaction effect on expectancy ratings (all *P* > 0.37; see Supplementary Table 2 for details). For a detailed description of the baseline memory performance and arousal ratings, see Supplementary Materials and Supplementary Tables 1–3.

### Oscillatory correlates of successful memory cueing during sleep

We focused on the analysis of oscillatory activity in the theta and spindle range associated with successful memory cueing during sleep based on our previous findings during NREM sleep^45^,^48^. We defined successful memory cueing as the difference between oscillatory responses for verbal cues presented during sleep for which the associated picture was later remembered vs. forgotten (subsequent cueing effect, SCE). Building upon those prior findings mentioned above, induced theta power (5–8 Hz) was analyzed between 300 and 900 ms, induced spindle power (12–15 Hz) between 800–1200 ms. First, we analyzed whether we could replicate our previous findings in the NREM group, before then extending the analysis to REM sleep.

**Figure 3:**
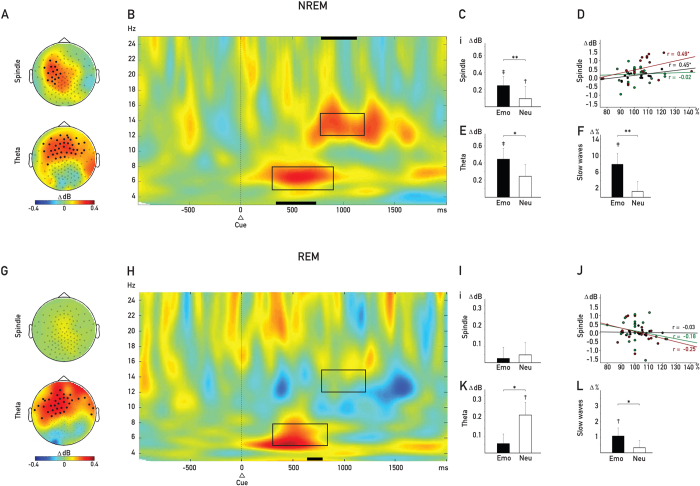
Oscillatory Results. Oscillatory activity after word replay (dotted line in (**B** and **F)**) was analyzed for *subsequently remembered* and *subsequently not remembered* trials in the theta (5-8 Hz) and spindle band (12–15 Hz). **NREM group** (**A**–**F**): (**A**) Significant clusters of electrodes. (**B)** Time frequency plot for the overlap of significant electrodes of the theta and spindle cluster. Differences in power were analyzed in the time window 300–900 ms for theta and 800–1′200 ms for spindle activity. For both, spindle (**C**) and theta (**E**), power was significantly stronger for *remembered* as compared to *not remembered* trials (ΔdB). The increased power for both was significantly more pronounced in emotional as compared to neutral trials. The increase in spindle activity (**D**) after cueing of *remembered* (black dots) trials correlated significantly with improved memory performance in the cued recall test. The correlation was stronger for emotional (red dots) and did not reach significance for neutral trials (green dots). The cues of *remembered* trials were followed by more slow waves **(F)** as compared to *not remembered* trials (represented by zero). Within the remembered trials, slow waves occurred more often in emotional as compared to neutral trials. In the **REM group** (**G**–**L**), power in the theta band for *remembered* trials was increased for the same time and frequency window (**H**) over the same cluster as in the NREM group (**G**). The enhanced power the theta band (**K**) for stronger for *remembered* as compared with *not remembered* (ΔdB) and more pronounced in *remembered* neutral as compared with *remembered* emotional trials. Spindle power (**I**) did not differ between the conditions in the REM group, and the increase did correlate with memory performance (**J**). Only *remembered* emotional trials were followed by more slow waves (**L**) as compared to *not remembered* trials (represented by zero) and also more often compared to neutral trials. The slow wave score was created by first calculating the percentage of trials followed by a slow wave, separately for *remembered* emotional and neutral and *not remembered* trials. Following, the percentage for *not remembered* was subtracted from *remembered* trials. *Values are means* ± *SEM*. *,†*P* < 0.05, **,‡*P* < 0.01.

As expected, we observed a significant SCE for both theta and spindle activity during NREM sleep: Theta power was significantly higher after verbal cues for which the associated pictures were later remembered vs. forgotten in a bilateral frontal electrode cluster (*P* = 0.014, corrected for multiple comparisons, see Fig. 3A (lower panel) and B). Similarly, several electrodes that were mostly left lateralized over central regions showed a positive SCE for induced spindle power (*P* = 0.04, corrected for multiple comparisons, see Fig. 3A (upper panel) and B). This two electrode clusters were used as region of interests (ROIs) for all subsequent analyses.

In a second step, we extracted oscillatory power within the respective ROIs for later remembered emotional associations and later remembered neutral associations separately. The later forgotten category was not divided by arousal due to a low number of trials in the emotional category. During NREM sleep, theta and spindle oscillatory power nicely reflected the behavioral result pattern with respect to cued recall performance: We observed a significantly higher response in both spindle and theta activity after later remembered cues that were associated with emotionally arousing pictures as compared to neutral pictures (theta: *t*_*19*_ = 2.20, *P* = 0.040, 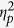 = 0.02; spindle: *t*_*19*_ = 2.69, *P* = 0.014, 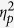 = 0.04, see Fig. 3C). In addition, while cueing of later remembered emotional associations induced a significant increase in theta (*t*_*19*_ = 2.85, *P* = 0.010, 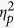 = 0.02) and spindle activity (*t*_*19*_ = 3.94, *P* = 0.001, 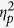 = 0.06) as compared to subsequently forgotten associations, this power increase for remembered was only observable in spindle (*t*_*19*_ = 2.25, *P* = 0.037, 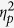 = 0.03) but not theta range (*t*_*19*_ = 1.22, *P* = 0.22; see Fig. 3C,E and Supplementary Fig. 2A). The improvement in overall cued recall performance in the NREM sleep group was positively correlated with cueing induced spindle power (*r* = 0.45, *P* = 0.04, Fig. 3D) but not with theta power (*r* = −0.03, *P* = 0.82, not shown) after cueing. Fitting well with the previous findings, the correlation was more robust for emotional (*r* = 0.49, *P* = 0.01) and was not significant for neutral trials (*r* = −0.02, *P* = 0.921: see Fig. 3D for spindle power). A direct comparison revealed that the correlation was significantly higher for emotional as compared to neutral associations (*P* = 0.047).

During REM sleep oscillatory responses for cues for which the associated picture was later remembered vs. forgotten differed only in the theta but not spindle range (see Fig. 3I,K). When extracting power values using the same (NREM) ROI, we found significantly increased theta activity for later remembered associations (*t*_*16*_ = 1.93, *P* = 0.07, 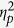 = 0.05). However, in contrast to the behavioral result pattern observed by cueing during REM sleep, verbal cues associated with later remembered neutral pictures induced the strongest increase in theta activity (*t*_*16*_ = 2.54, *P* = 0.022, 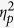 = 0.15), whereas induced theta activity for cues associated with emotionally arousing pictures did not differ from non-remembered trials (*t*_*16*_ = 0.83, *P* = 0.42, see Fig. 3K). In fact, theta power was even significantly smaller for later remembered emotional as compared to neutral associations (*t*_*16*_ = −2.61, *P* = 0.019, 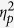 = 0.06). Concerning activity in the spindle range during REM sleep, we did not observe any differences between these trials types for the (NREM-) ROI (see Fig. 3I) and no significant correlations with memory performance were observed during REM sleep (Fig. 3J). Analyzing the oscillatory correlates of successful memory cueing also during REM sleep alone revealed the same pattern of results, only in a slightly smaller cluster (for a detailed description, see Supplementary Materials).

As slow oscillations have been suggested to play a functional role in consolidating reactivated memories during sleep^6^, we counted cue presentations that were followed by surface slow waves at frontal electrode sites. Slow waves were defined as waves with a duration of more than 500 ms, exceeding an amplitude of 75 μV and initiating between 0 and 800 ms after stimulus onset. As reported previously^45^,^48^, we observed that the occurrence of slow waves was higher after cueing of later remembered as compared to later not remembered associations during NREM sleep (*t*_*19*_ = 4.36, *P* < 0.001, 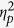 = 0.57, see Fig. 3F). Again paralleling the pattern of results of the behavioral and oscillatory analyses in the NREM sleep group, cueing of later remembered emotional associations was followed by more slow waves as compared to later remembered neutral associations (*t*_*19*_ = 3.01, *P* = 0.007, 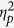 = 0.14). Remarkably, we also observed a higher number of slow waves for later remembered as compared to forgotten associations after cueing during REM sleep independent of arousal (*t*_*16*_ = 2.28, *P* = 0.036, 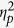 = 0.03). In addition, while cueing of later remembered emotional associations was associated with a higher number of slow waves (*t*_*16*_ = 2.07, *P* = 0.055, 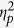 = 0.07), cueing of later remembered neutral associations was not (*t*_*16*_ = 0.74, *P* = 0.492, 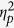 = 0.01), and this difference was significant (*t*_*16*_ = 3.06, *P* = 0.031, 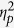 = 0.07, see Fig. 3 and Table 2 for details). Note that REM sleep was scored in spite of cue-elicited slow waves (see methods). Thus, cueing success during REM sleep might a least partly depend on successful triggering of slow waves by the cue.

### Analyses of subcategories

In an exploratory analysis, we examined whether a more fine-graded categorization of emotionality provides further support of the modulation of cueing effects by arousal levels and sleep stages. The emotional arousal for each picture was defined by individual ratings acquired prior to the associative learning task, and the emotional and neutral picture category were each divided into two equally sized sub-sets. Within the emotional pictures, the 25 pictures rated with the highest emotional arousal were classified as emotionally high (Emotional+) and the remaining 25 pictures as emotionally low arousing (Emotional−). The same subdivision was applied for the neutral pictures, resulting in the categories neutral high (Neutral+) and neutral low arousing (Neutral−; see Supplementary Fig. 3 for a representative picture of each category).

**Figure 4:**
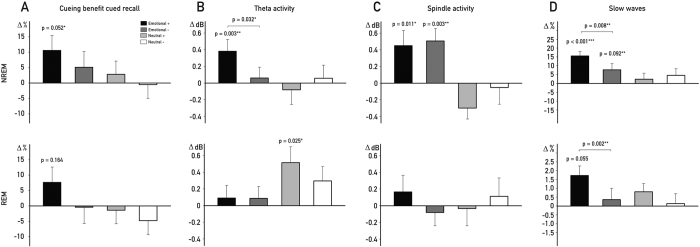
Cued Recall Power Changes in the four Subcategories. Differences in cueing benefits, oscillatory activity in the theta (5–8 Hz) and spindle range (12–15 Hz) and slow waves in the NREM and REM group after splitting each, the emotional and neutral category into a high (*Emotional*+ and *Neutral*+, respectively) and low arousing subcategory (*Emotional*− and *Neutral*−, respectively). Cueing benefits ((**A)** upper panel) were limited in the NREM group to emotionally high arousing (*Emotional*+) and not observable for emotionally low arousing associations (*Emotional*−). Mirroring this effect, the increase in theta activity for *remembered* trials ((**B)** upper panel) was mainly driven by the same category but spindle activity ((**C**) upper panel) was generally increased for remembered emotional association. Cueings of *subsequently remembered* Emotional+ were more often followed by slow waves as compared to Emotional− trials. A trend towards a significant cueing benefit (**A** lower panel) emerged also in the REM group (*P* = 0.16) and was restricted to the emotionally high arousing subcategory. Theta power was generally more pronounced in *remembered* neutral as compared with *remembered* emotional trials but the neutrally high (*Neutral*+) and low arousing trials (*Neutral*−) did not differ ((**B)** lower panel). Spindle power **((C)** lower panel) was not significantly different for any subcategory and did not differ between the four categories in the REM group. Successfully cued Emotional+ were more often followed by slow waves as compared to subsequently remembered Emotional− trials ((**D)** lower panel). The cueing benefit score was created by first calculating the change of correctly recalled associations for cued and uncued associations separately, with setting the performance before the retention interval to 100%. Following, the uncued was subtracted from the cued score. Values are mean ± SEM. *P ≤ 0.05 **P ≤ 0.01 ***P ≤ 0.001.

The cueing benefit during NREM sleep within the emotional category was mainly driven by memories for Emotional+ (10.61 ± 3.12%; *t*_*1,20*_ = 2.06, *P* = 0.052, 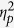 = 0.09, see Fig. 4A, upper panel) and not significant from zero for Emotional− (6.51 ± 3.87%; *t*_*1,20*_ = 0.92, *P* = 0.368). Theta activity was also only significantly enhanced for Emotional+ (*t*_*19*_ = 2.31, *P* = 0.003, 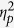 = 0.08) and more pronounced for later remembered Emotional+ vs Emotional− (*t*_*19*_ = 2.31, *P* = 0.032, 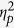 = 0.08), mirroring the behavioral findings (see Fig. 4B, upper panel). However, spindle activity during NREM sleep was significantly enhanced for both Emotional+ and Emotional− pictures (both *P* < 0.02) and did not differ between the two emotional categories (*P* = 0.69; see Fig. 4C, upper panel). Later remembered Emotional+ were also more often followed by slow waves as compared to subsequently not remembered (*t*_*19*_ = 6.21, *P* < 0.001, 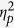 = 0.34; see Fig. 4D upper panel) and as compared to Emotional− (*t*_*19*_ = 3.92, *P* = 0.008, 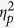 = 0.17).

Interestingly, analyzing the extreme category also in the REM sleep group, revealed that cueing the most arousing associations (Emotional+) improved memory significantly as compared to waking (*P* = 0.04), however the cueing benefit was not significantly different from zero (*P* = 0.16, see Fig. 4A, lower panel). While it seems that more intense associations tend to benefit from cueing also during REM sleep, the difference between Emotional+ and Emotional− was not significant (7.66 ± 5.01% vs −0.41 ± 5.27%: *P* = 0.33). Theta activity was significantly increased for the Neutral+ pictures only, while no increases were observed for spindle activity during REM sleep for any category (see Fig. 4B and C, lower panel). Mirroring the cueing benefit, only successfully cued Emotional+ (*t*_*16*_ = 6.21, *P* = 0.005, 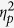 = 0.12; see Fig. 4D lower panel) and not Emotional− associations (*t*_*16*_ = 0.56, *P* = 0.579, 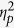 = 0.01) were more often followed by slow waves as compared to subsequently not remembered trials.

## Discussion

For the first time, our study directly contrasted effects of auditory cueing of emotionally arousing memories during deep NREM and REM sleep as well as wakefulness. Contrary to our hypothesis, we did not find any hint for a preferential and selective cueing benefit during REM sleep for emotional memories. In fact, cueing memories during NREM sleep resulted in a robust preferential strengthening of emotional as compared to neutral memories. Further corroborating the emotional modulation of cueing, only highly arousing emotional memories benefitted from cueing with a gradual decline of cueing benefit with decreasing arousal. A similar but weaker effect for highly arousing memories was observed during REM sleep, which, however, did not reach statistical significance. During REM sleep might only highly disturbing memories are re-processed and hence consolidated. Future studies, using exclusively stimuli eliciting strong emotional arousal possibly strengthen the effect of cueing during REM sleep. Consistent with previous studies, we did not observe any cueing benefits when cues were presented during post-learning wakefulness. Cues were presented during “active waking”, i.e., participants performed on a working memory task while the cues were deliver. As we have repeatedly shown that effects of cueing during wakefulness do not differ between passive and active waking conditions^45^,^56^, we can exclude that the working memory task interfered with the processing of the memory cues.

Our results for a benefit of cueing emotional memories during NREM sleep are in line with the notion that replay during NREM sleep is modulated by emotionality and future relevance of newly encoded stimuli, leading to increased replay of emotionally salient and rewarded stimuli during NREM sleep and improved later recall^13^,^46^,^57^,^58^. However, our results are not in line with the view that reactivations during REM sleep are functionally relevant for the assumed role of REM sleep in the preferential consolidation of emotional memories, particularly as compared to NREM sleep.

Our results from the oscillatory analyses support this conclusion. According to our working model derived from our previous studies with (neutral) vocabulary cueing during sleep^59^, induced theta oscillations reflect the successful reinstatement of the memory trace by cueing. Subsequently, induced spindle oscillations are mandatory for a successful stabilization and strengthening of the memory trace after cueing. The role of sleep spindle oscillations in memory consolidation and plasticity is widely supported^6^. Specifically, hippocampal signals of reactivation are assumed to be nested in individual troughs of spindles^60^,^61^,^62^. Furthermore, sleep spindles are thought to prime and maintain long-term potentiation in cortical circuits by provoking Ca^2+^ influx for successive plasticity associated processes^63^, possibly thereby supporting the redistribution of reactivated memories to cortical long-term memory stores^64^.

Unlike spindle power, the involvement of theta activity during NREM sleep for memory consolidation is not yet widely accepted. During wakefulness, theta activity is consistently associated with memory formation and function in a number of species and predicts success of later recall^65^,^66^. Furthermore verbal cueing during sleep leads to enhanced theta activity during later recognition testing, possibly indicating the presence of a stronger memory trace^67^. It is assumed that theta activity is crucial for binding information together, particularly binding information from disparate brain regions during encoding and retrieval^68^,^69^. During sleep, increased theta power is also associated with better memory consolidation in healthy participants and patients^70^,^71^. In the present study, successful cueing of emotional and neutral pictures during NREM sleep elicited both increased theta and spindle activity, replicating our previous finding from vocabulary learning also in terms of timing and topography^45^,^48^. Importantly, the subsequent cueing effect (SCE) during NREM sleep differed solely quantitatively, but not qualitatively between emotionally arousing and neutral memories: Successful cueing of emotional pictures revealed a stronger increase in theta and spindle oscillations as compared to neutral pictures, thereby paralleling the improvements by cueing for emotional vs. neutral pictures on the behavioral level. In addition, the increase in induced spindle power was positively correlated with the individual retention performance.

In contrast, during REM sleep, we did not observe increased theta activity during the successful cueing of emotional memories or the subcategory of memories eliciting a strong arousal, in spite of some descriptive hints for behavioral improvements after cueing during REM sleep. For neutral memories, an increase in theta oscillations was observed during REM sleep without any improvements on the behavioral level. Furthermore, cueing during REM sleep did not induce activity in the spindle range, neither for emotional nor neutral memories. Evidence that auditory stimuli are processed during REM sleep comes from the observation that dream content can be modified by presenting words during REM sleep^72^,^73^. In spite of this successful reinstatement by presenting memory cues during this sleep stage, cued memories are not stabilized during REM sleep, probably because of the missing spindle activity and associated plastic processes.

On the behavioral level, we did not find any effect of TMR during REM sleep on arousal ratings. This contradicts the assumption that replay of emotional memories during REM sleep affects the arousal component of emotional memories as stated by the sleep-to-remember – sleep-to-forget hypothesis^30^. In addition, we did not find any influence of TMR during REM and NREM sleep on expectancy ratings, a measure typically used in fear conditioning research^74^. It remains, however, unclear whether these findings also apply for traumatic memories and studies with a stronger clinical focus are needed. For cued recall testing, we observed a clear benefit of cueing during NREM sleep for emotionally arousing memories, while the cueing benefit did not reach statistical significance for neutral memories. However, several previous studies have in fact reported significant increases in recall performance after cueing during NREM sleep for neutral memories, including spatial and verbal memories^41^,^59^. Interestingly, sleep-dependent memory consolidation of neutral memories can become attenuated when emotionally arousing items are included into the learning material^22^,^28^,^75^,^76^. Thus, it might be possible that the preferential reactivation of emotionally memories during NREM sleep biases the sleep-dependent consolidation processes. In other words, sleep and cueing benefits for neutral memories might be reduced when emotionally arousing memories are learned concomitantly. However, this notion requires further testing.

Arguing against the notion of a distinct role of REM sleep for emotional memories, it has been repeatedly suggested that REM sleep might serve distinct but complementary functions for memory consolidation^6^. For example, Llewellyn & Hobson^77^ argue that REM memory processing cannot be considered in isolation from the role of NREM and Giuditta and colleagues^78^ suggest that the optimum benefits of sleep on the consolidation of both declarative and non-declarative memory occur when NREM and REM sleep take place in succession. While during NREM sleep previously acquired memories are reactivated and reorganized, REM sleep then might function to support processes of synaptic plastic changes, creating enduring connections for the long-term. Thus, it might be possible that emotionally arousing memories are preferentially replayed during NREM sleep, and then additional memory processes occur during subsequent REM sleep which do not involve replay of memories (or are at least less influenced by targeted memory reactivation).

Another interpretation of our findings is that cueing during REM sleep does not lead to a strengthening of the exact same memory trace, but lead to other memory processes involving generalization or strengthening of previously weaker, more “far-fetched” associations. While cueing during REM sleep had no effect on accurate memory performance in the study by Sterpenich and colleagues^54^, it increased the number of false positives. In addition, Stickgold and colleagues^79^ propose that weaker associations are replayed during REM sleep, possibly playing a role in creative processes. Interestingly, we observed stronger theta oscillatory power after cueing of neutral compared to emotional memories, in spite of a trend towards a better (accurate) memory recall for emotionally high arousing memories. This “inverse” theta effect possibly reflect a higher success of cueing several rather “far-fetched” memory associations, which are less relevant for accurate remembering but involved in creative processes.

A third alternative interpretation of our findings is that targeted memory reactivations have indeed no effect on memory processes during REM sleep, and that the small but non-significant improvements reported here are due to NREM-like processes occurring during REM sleep. For example, a recent study in mice demonstrated that slow waves, the hallmark of NREM sleep, occur regularly also during REM sleep but only in deeper layers of primary cortical areas^80^, which go undetected in recordings from the scalp. A similar finding has been reported from studies in rats (Vyazovskiy, personal communication). In addition, we observed that several successfully cued memories induced slow waves or K-complexes during REM sleep (note that REM scoring was only continued after the occurrence of an isolated K-complex following the word presentation when (a) the remaining epoch met all criteria for scoring of REM sleep, (b) rapid eye movements were visible in the same epoch and (c) both the previous and subsequent epoch were unequivocally scored as REM sleep). Thus, it might be possible that targeted memory reactivation during REM sleep recruited (or induced) some NREM sleep like features, which resulted in a weak improvement of emotional memories by cueing. Interestingly, Funk and colleagues^80^ did not detect sleep spindles during REM sleep. This is consistent with our finding that activity in the spindle range is involved in memory processing only during NREM but not REM sleep. According to this interpretation, targeted memory reactivation for both, emotional and neutral memories, is only beneficial when cues are either presented during NREM sleep or when they involve NREM-like features during REM sleep.

Taken together, our findings provide no evidence for the notion that cueing during REM sleep explains a preferential consolidation of emotionally arousing memories during this sleep stage. In contrast, emotional arousal during encoding modulated oscillatory correlates during NREM in a quantitative rather than qualitative manner, possibly indicating that emotional and neutral (declarative) memories are consolidated by similar mechanisms during NREM sleep. According to this “modulation hypothesis” of memory consolidation during sleep, emotion would result in a preferential replay and consolidation of memories during NREM sleep, which might underlie the observed cueing benefits and increased oscillatory correlates for emotional memories during NREM sleep reported here. Whereas during REM sleep, the occurrence of NREM-like features (i.e. slow waves etc.) might be necessary for inducing memory benefits by targeted memory reactivation during sleep.

## Materials and Methods

### Participants

Sixty-two subjects participated in the three experimental groups (NREM sleep group: n = 21, REM sleep group: n = 20 and Wake group: n = 21, see Table 3 for details). Participants were free of any medication at the time of the experiment and none had a history of any neurological or psychiatric disorders. All subjects reported a normal sleep-wake cycle and none had been on a night shift for at least eight weeks before the experiment. On experimental days, subjects were instructed to get up at 7.00 h and were not allowed to take in caffeine and alcohol or to nap during daytime.

The study was approved by the ethics committee of the Department of Psychology, University of Zurich, and all methods were performed according to the relevant guidelines and regulations. All subjects gave written informed consent prior to participating. After completing the whole experiment, participants received 120 Swiss francs (CHF) (sleep groups) or 100 CHF (wake groups), respectively.

### General procedure

Participants entered the laboratory at 9 pm. The session started with the application of the electrodes for standard polysomnography, including electroencephalographic (EEG; 128 channels, Electrical Geodesic, Inc.), electromyographic (EMG), and electrocardiographic (ECG) recordings. Prior to the experiment, participants of the sleep group spent an adaptation night in the sleep laboratory.

In all three experimental groups, the learning phase started at ~10 pm with the associative emotional memory task (for details see *The associative emotional memory task*). After completing the learning task, participants of both sleep groups went to bed at 11 pm and were allowed to sleep for 3 h (NREM group) or 6 h (REM group), respectively whereas participants in the wake control groups stayed awake during 3 h (see Fig. 1, for an overview of the procedure). During the retention interval, a selection of prior learned words was presented for ~80 minutes during sleep stages N2 and N3 for participants of the NREM group, during stage REM for participants of the REM sleep group and during wake for participants of the wake group, respectively (see below for a detailed description of the cueing phase).

### Learning tasks

#### The associative emotional memory task

The associative emotional memory task consisted of 100 words and pictures, which were grouped into association pairs. The words were two syllable substantives selected from the Berlin Affective Word List Reloaded^81^, a German database providing normative ratings for emotional valence and arousal. The selection criterion was a neutral rating with a very small standard deviation (mean rating 0 ± 0.2 SD on a −3 to +3 scale). In order to provide an auditory presentation, words were read by an actress and recorded in-house (sound file duration: 400–700 ms). The set of visual stimuli consisted of 100 pictures taken from the Nencki Affective Picture System^82^ (NAPS). Fifty of these pictures are generally rated as neutral and low arousing. The remaining 50 pictures elicit a high level of emotional arousal and are perceived as negative in emotional valence. The learning material for a single subject consisted therefore of 100 neutral words of which 50 words were associated with emotionally arousing pictures and the remaining 50 words were associated with neutral pictures. The associations between words and pictures were balanced across participants. Since the perception of pictures as arousing can differ dramatically between subjects, we further subdivided the emotional picture set based on individual arousal ratings into two equally sized categories. This resulted in a set of pictures comprising the 25 most arousing pictures (“emotional high arousing”) and a picture set comprising the 25 less arousing emotional pictures (“emotional low arousing”). The same procedure was applied for the neutral pictures, resulting in the two sub-sets “neutral high arousing” and “neutral low arousing”.

#### Arousal ratings

Prior to the associative emotional memory task, participants rated words and pictures separately on a 7-point Likert-scale with respect to the level of arousal, ranging from ‘not at all arousing’ to ’highly arousing’. Following a fixating cross, displayed for 1,000 ms in the center of the screen, the respective word was presented auditory via head-phones. Subsequently, the rating scale was displayed and subjects were instructed to use the keyboard to indicate the arousal elicited by the stimulus. The rating of the picture followed the same procedure, with pictures being presented on the computer screen for 1,500 ms.

Participants of the study rated emotional pictures as more arousing than neutral pictures (5.31 ± 0.08 vs. 2.23 ± 0.13; F_1,59_ = 829, p < 0.001, η^2^ = 0.753) with no significant difference between the three groups (F_2,59_ = 0.113, p = 0.87).

#### The learning phase

During the associative emotional learning paradigm, participants were instructed to memorize the word-picture pairs. They had to complete a total of three rounds: During the first round, the words were presented auditory after a fixation cross (1,000 ms), followed by visual presentation of the picture on the computer screen. 1,000 ms after picture onset, the respective word was played a second time. The picture was presented for 2,500 ms followed by an inter-stimulus interval (ISI) with a random duration (1,000–2,000 ms). No response was required during the first learning round. During the subsequent two rounds, the first word presentation was again followed by a 7-point Likert-scale and participants had to indicate whether they expected a low or high arousing picture. Subjects were instructed to press ‘7’ (or ‘1’), if they expected a high (or low, respectively) emotionally arousing picture, with highest certainty. The “medium” button ‘4’ indicated no expectancy, while the remaining buttons were used for graded levels of confidence. Participants were instructed to avoid guessing and to rely on their feelings of expectancy, rather than on explicit memory for the picture. In any case, following the key press the correct picture was presented again for 2000 ms, serving as feedback.

After a delay of 15 min, subjects underwent two types of recall testing. Using the same procedure as during the learning procedure, subjects had to indicate the expectancy of an arousing or non-arousing picture but no feedback was provided in form of the associated picture. Therefore, the response served as an index for successful implicit memory formation. In contrast to the cued recall test, expectancy ratings require only the anticipation of an event and no explicit or detailed description is needed. Traditionally, this type of learning test is used in the fear conditioning research as a verbal measure of the extent to which participants expect the unconditioned stimulus upon presentation of the conditioned stimulus^83^,^84^. The acquisition of information conveyed by expectancy is attributed to associative learning process of classical conditioning^74^.

Declarative memory performance was tested using a cued recall procedure: Each trial started with a fixation cross signalizing the auditory presentation of the word. If participants were able to remember the associated picture they were instructed to press ‘Y’ and subsequently type a short description of the content of the picture. If they did not remember the associated pictures, they pressed ‘N’ and the next word was presented. Retrieval performance was tested twice: following learning (baseline) and after the retention interval (final retrieval). As retention score we calculated the percentage of final retrieval performance with retrieval performance at baseline set to 100%.

#### Cueing of associations during sleep

After learning, participants of the NREM sleep group slept for 3 hrs whereas the wake group had to stay awake during the same period and participants of the REM sleep group were allowed to sleep for 6 hrs. The rationale behind the different sleep durations for the NREM and REM sleep group is derived from the observation that the first night half is dominated by NREM sleep, while REM sleep is more dominant in the second night half. Cueing was initiated following online detection of stable NREM (four consecutive epochs of N2 or N3) or REM sleep (all criteria for REM scoring met and rapid eye movements visible) and terminated immediately when arousal or polysomnographic signs of sleep stage changes occurred. Sleep was continuously monitored by the experimenter. In the wake group, cueing of associations occurred during performance of a computerized n-back task. The 3-h wake retention interval was divided into 30-min periods. During the first, third and fifth period, participants performed on the n-back task (including a total of 27 67-s blocks of 0-back, 1-back and 2-back, see task description for details). Participants were instructed to focus on the task and accuracy was monitored after each 30-min period.

During the retention interval, half of the words of the associations were repeatedly presented aurally via loudspeakers (50-dB sound pressure level). For each participant, the words were selected for cueing based on the last expectancy ratings. For each certainty category an automatic MATLAB algorithm chose randomly half of the words, resulting in a total of 50 words. Presentation occurred every 8,000–8,200 ms in a randomized order during 80 min (see Table 1 for sleep parameters and number of cueing).

### n-back task

Subjects of the wake group performed on 0-, 1- and 2-back versions of n-back working memory task^85^. In this task, subjects are presented with a continuous stream of letters and are instructed to press a key whenever the letter ‘x’ occurs (0-back), or when they detect a repetition at a specified delay. In the 1-back version, subjects have to respond to immediate letter repetition (t-h-v-v) and in the 2-back version to a letter repetition with one intervening letter (t-h-v-h).

### Sleep EEG

Sleep was monitored and recorded via a standard polysomnography electrode montage including EEG, EOG, submental EMG and ECG (AASM). High-density EEG (128 channel Geodesic Sensor Net, Geodesics, Eugene, OR, USA) was used to reliably estimate the topographical distributions of neuronal effects related to the cueing. Impedances were kept below 50 kΩ, while signals were sampled online at 500 Hz and referenced to the vertex electrode (Cz). In addition to the online identification of sleep stages, polysomnographic recordings were scored offline by two independent raters according to standard criteria^86^ and discrepant scorings were solved with the aid of a third rater. In case the word presentation during REM sleep elicited an isolated K-complex but all criteria for REM scoring were met in the current, the previous and subsequent previous and rapid eye movements were visible in the same epoch, the scoring of REM sleep was continued.

### Oscillatory analysis

EEG signals were preprocessed using Brain Vision Analyzer 2.0 (Brain Products, Gilching, Germany). Initially, raw EEG data were re-referenced to the average of the two mastoids and as well low-pass (100 Hz, roll-off 12 dB per octave) and high-pass filtered (0.4 Hz, roll-off 12 dB per octave). Before categorization into emotional and neutral trials, sleep EEG data were epoched into segments ranging from 1,000 ms before to 2,500 ms after word onset and trials with artifacts, e.g. due to movements, were removed after visual inspection. REM segments were additionally corrected for eye movements using the Gratton and Coles algorithm^87^. Subsequently, segments were categorized in emotional and neutral later remembered and later forgotten associations, respectively. Three subjects from the REM and one subject from the NREM sleep group had to be excluded from the oscillatory analysis due to technical problems during data acquisition.

All succeeding EEG oscillatory analyses steps were performed using the open source Fieldtrip toolbox^88^ (http://www.fieldtriptoolbox.org) running on Matlab R2012b (MathWorks, Natick, MA). First, the data segments were averaged (evoked response) and then subtracted from the time-domain EEG signal on each trial. This was accomplished separately for each condition, electrode, and subject. The evoked (non-phase-locked) response was subtracted to isolate induced oscillations, which are thought to be generated by high-order processes^89^,^90^.

Time-frequency analysis was computed for each trial by using a 7-cycle Morlet wavelet decomposition, ranging from 2 to 30 Hz in 0.5 Hz steps. A sliding window with a step size of 50 ms was applied across the entire length of the epochs. Subsequently power estimates were decibel normalized (dB power = 10*log10(power/baseline)), using a baseline window ranging from −1,000 ms to −100 ms before stimulus onset.

### Slow wave analysis

The EEG segments of later remembered emotional arousing, neutral and later not remembered trials (−2000 to 4500 ms) were band-pass filtered in the range of 0.5–4 Hz. Slow waves were detected by a rater blind to the categories at electrode site Fz, F3 and F4 and defined as waves with a duration of more than 500 ms, exceeding an amplitude of 75 μV and initiating between 0 and 800 ms after stimulus onset.

### Statistical analysis

Behavioral data were analyzed using repeated analyses of variance (ANOVA) including the within-subject factor ‘emotional arousal’ (emotional vs. neutral) and the between-subject factors ‘sleep/wake’ and ‘group’ (NREM, REM and Wake). Post-hoc analyses were conducted using *t*-tests for pair-wise comparisons and Pearson correlations. A probability of *P* = 0.05 was set as significance threshold. For analyzing the cued recall performance we calculated separately for cued and uncued associations the changes in cued recall performance as percentage of correctly recalled associations with performance before the retention interval set to 100%. Subsequently, a *cueing benefit* score was created by calculating the difference between the memory performance of cued and uncued associations.

Statistical analyses of the EEG data was performed with a nonparametric randomization test using cluster correction^88^ as implemented in FieldTrip. Statistics were performed on time segments ranging from stimulus onset to 2.000 ms. The cluster alpha was set to 0.05 and 1000 randomizations were conducted for all tests. Clusters were considered significant at P < 0.05 (two-sided).

## Additional Information

**How to cite this article**: Lehmann, M. *et al*. Emotional arousal modulates oscillatory correlates of targeted memory reactivation during NREM, but not REM sleep. *Sci. Rep.* **6**, 39229; doi: 10.1038/srep39229 (2016).

**Publisher's note:** Springer Nature remains neutral with regard to jurisdictional claims in published maps and institutional affiliations.

## Supplementary Material

Supplementary Material

## Figures and Tables

**Table 1:** Sleep and cueing parameter.

	NREM	REM	*t*	*P*
*Sleep Parameters*				
*Total Sleep Duration (min*)	194.64 ± 4.17	354.82 ± 4.63	−25.70	**<0.001**
* N1 (%*)	5.19 ± 0.62	2.90 ± 0.47	3.03	**0.005**
* N2 (%*)	47.22 ± 2.26	47.94 ± 2.35	−0.18	0.863
* N3 (%*)	30.74 ± 2.30	27.91 ± 2.59	0.70	0.492
* REM (%*)	13.83 ± 2.30	20.31 ± 1.03	−3.67	**0.001**
* WASO (%*)	2.01 ± 1.33	0.33 ± 0.15	1.59	0.122
*Number of cueings*				
* Emotional*	281.63 ± 5.12	212.89 ± 6.02	5.56	**0.001**
* Neutral*	267.29± 4.78	216.332 ± 7.27	3.12	**<0.01**

WASO = wake after sleep onset. *Data are means* ± *SEM*.

**Table 2:** Slow waves after cueing.

		Later remembered	Later not remembered	*t*	*P*
***NREM***	*All*	50.49 ± 2.19	44.16 ± 2.61	4.36	**<0.001**
*Emotional*		53.93 ± 2.45	44.16 ± 2.61	5.60	**<0.001**
*Neutral*		49.46 ± 2.21	44.16 ± 2.61	3.49	**0.002**
***REM***	*All*	3.43 ± 0.62	1.93 ± 0.43	2.29	**0.036**
*Emotional*		4.34 ± 0.78	1.93 ± 0.43	3.42	**0.004**
*Neutral*		3.11 ± 0.65	1.93 ± 0.43	1.62	0.125
		**Emotional remembered**	**Neutral remembered**		
***NREM***		53.93 ± 2.45	49.46 ± 2.21	3.13	**0.005**
***REM***		4.34 ± 0.78	3.11 ± 0.65	1.85	0.083

Values indicate percentage of word presentations during NREM and REM sleep that were followed by slow waves within 800 ms after word onset. *Data are means* ± *SEM*.

**Table 3:** Demographic data.

	*N*	Age	m/f
***NREM***	*21*	22.1 ± 0.5	5/16
***REM***	*20*	22.3 ± 0.8	4/16
***Wake***	*21*	23.5 ± 0.6	5/16

Demographic data of the three experimental groups.
