# Supplementary material for: Emotional arousal modulates oscillatory correlates of targeted memory reactivation during NREM, but not REM sleep
Source: Sci Rep. 2016 Dec 16;6:39229. doi: 10.1038/srep39229 (PMC5159847; doi:10.1038/srep39229)
Supplement: Supplementary Material [file srep39229-s1.pdf]

Supplementary Materials

Emotional arousal modulates oscillatory correlates of targeted memory reactivation during NREM, but not REM sleep

Mick Lehmann[1,2,3] Thomas Schreiner[3,4], Erich Seifritz[1,3] & Björn Rasch[3,5*]

[1] Department of Psychiatry, Psychotherapy and Psychosomatics, Psychiatric Hospital University of Zurich, Switzerland

[2] Institute of Psychology, University of Zurich, Zurich, Switzerland

[3] Zurich Center for Interdisciplinary Sleep Research (ZiS), University of Zurich, Switzerland

[4] Radboud University, Donders Institute for Brain, Cognition and Behaviour, Nijmegen, The Netherlands

[5] Department of Psychology, University of Fribourg, Fribourg, Switzerland

Correspondence and requests for materials should be addressed to B.R. (email: bjoern.Rasch@unifr.ch)

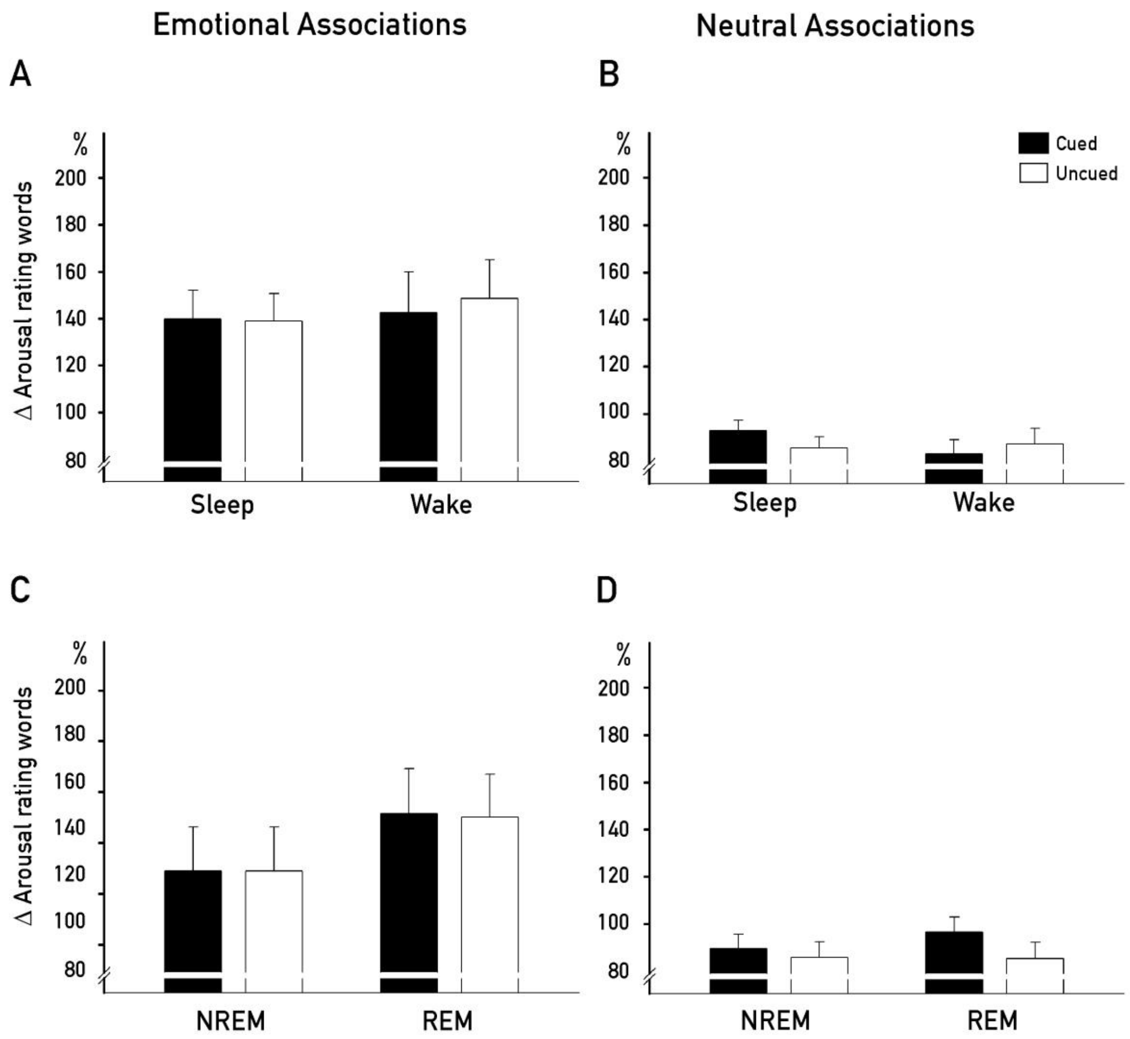

Supplementary Figure 1: Arousal Ratings

Changes in arousal ratings of neutral words. After the retention interval, the arousal ratings for words that were associated with emotional pictures **(A + C)** during the learning phase were rated overall as more arousing as compared to words with neutral picture associations **(B + D)**. **A + B** Changes in arousal ratings after sleep did not differ from changes after a retention interval filled with wakefulness. Furthermore, arousal ratings for words associated with emotional **(A)** or neutral pictures **(B)** were not affected by cueing during sleep or wakefulness. **C + D** Changes in arousal ratings did not differ between NREM and REM group and cueing did not change arousal ratings significantly. Changes in arousal ratings are indicated as percentage of ratings giving at the end of the experimental procedure, with arousal rated before the learning phase set to 100%. Values are mean ± SEM.

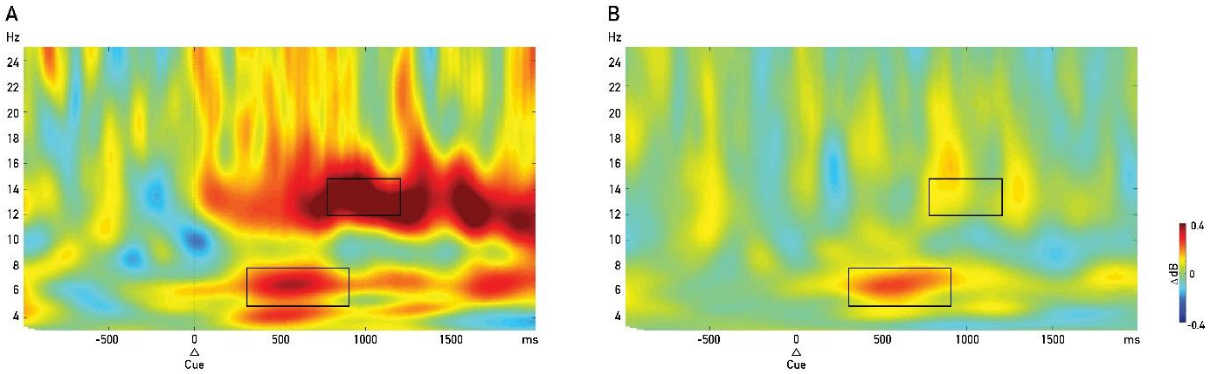

Supplementary Figure 2: Oscillatory analysis

Time frequency plot for oscillatory analysis in the theta (5-8 Hz) and spindle band (12-15 Hz) for emotional and neutral trials in the NREM sleep group. Oscillatory activity after word replay (dotted line in **A and B**) was analyzed for *subsequently remembered emotional* **(A)** and *subsequently remembered neutral trials* **(B)** and contrasted with *subsequently not remembered* trials. Differences in power of theta and spindle activity were statistically tested in the time window as illustrated with the black rectangles (theta band: 300-900ms and spindle band: 800-1’200ms) Theta and spindle power were significantly higher for *subsequently remembered emotional* **(A)** and *subsequently remembered neutral trials* **(B)** as compared to *subsequently not remembered trials*.

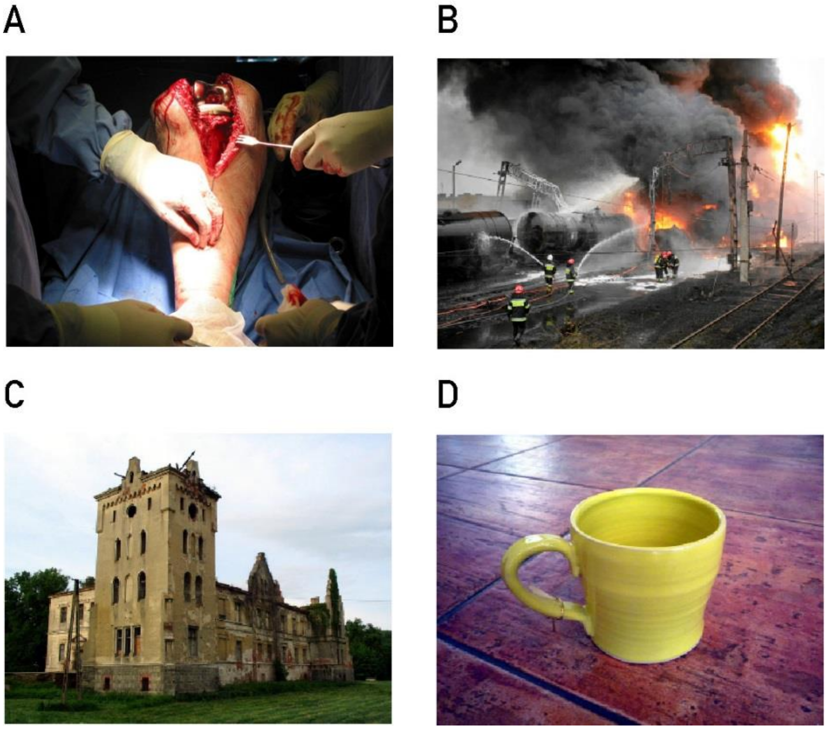

Supplementary Figure 3: Picture Examples

Representative examples of pictures from each category. Based on individual arousal ratings, the 50 emotional pictures were split into an emotionally high (Emotional + (**A**)) and an emotionally low arousing subcategory (Emotional- (**B**)). Accordingly, the 50 neutral pictures were split into Neutral+ (**C**) and Neutral- (**D**). Each category comprises 25 pictures.

Supplementary Table 1: Cued recall performance for categories 'Emotional' and 'Neutral'

		**Cued**	**Uncued**	***t***	***P***
**NREM**	Emotional				
	Learning	18.19 ± 0.81	18.24 ± 0.77	-0.77	0.942
	Retrieval	18.62 ± 0.74	17.67 ± 0.77	1.58	0.133
	Change	0.43 ± 0.51	-0.57 ± 0.33	2.11	**0.047**
	% Change	103.76 ± 3.08	97.26 ± 1.97	2.30	**0.032**
	Neutral				
	Learning	18.38 ± 0.78	18.43 ± 0.77	-0.12	0.912
	Retrieval	18.57 ± 0.78	18.43 ± 0.78	0.22	0.835
	Change	0.19 ± 0.34	0.00 ± 0.28	0.47	0.646
	% Change	101.67 ± 2.08	100.26 ± 1.63	0.61	0.553
	All				
	Learning	36.57 ± 1.46	36.67 ± 1.43	-0.14	0.891
	Retrieval	37.19 ± 1.29	36.09 ± 1.36	1.31	0.212
	Change	0.62 ± 0.63	-0.58 ± 0.38	2.13	0.046
	% Change	102.63 ± 2.15	98.71 ± 1.19	2.33	**0.030**
**REM**	Emotional				
	Learning	17.00 ± 0.75	17.55 ± 0.70	-1.05	0.313
	Retrieval	17.55 ± 0.94	17.50 ± 0.65	0.08	0.942
	Change	0.55 ±0.47	-0.05 ± 0.37	1.10	0.295
	% Change	102.84 ± 3.01	100.37 ± 2.16	0.79	0.449
	Neutral				
	Learning	16.40 ± 0.88	16.80 ± 0.92	-0.64	0.531
	Retrieval	16.35 ± 0.96	17.05 ± 0.92	-1.51	0.154
	Change	-0.05 ± 0.34	0.25 ± 0.26	-0.66	0.515
	% Change	99.34 ± 2.49	101.79 ± 1.69	-0.75	0.465
	All				
	Learning	33.40 ± 1.52	34.35 ± 1.50	-1.02	0.325
	Retrieval	33.90 ± 1.77	34.55 ± 1.45	-0.82	0.427
	Change	0.50 ± 0.60	0.20 0.47	0.42	0.686
	% Change	101.05 ± 2.10	101.00 ± 1.54	0.02	0.982
**Wake**	Emotional				
	Learning	18.10 ± 0.90	18.95 ±0.70	-1.44	0.173
	Retrieval	17.48 ± 0.98	19.14 ± 0.85	-2.97	0.014
	Change	-0.62 ± 0.45	0.19 ± 0.44	-1.80	0.092
	% Change	96.59 ± 2.73	100.90 ± 2.27	-1.85	0.084
	Neutral				
	Learning	19.00 ± 0.60	17.57 ± 0.78	2.32	**0.033**
	Retrieval	18.48 ± 0.65	16.76 ± 0.85	2.89	**0.014**
	Change	-0.52 ± 0.46	-0.81 ± 0.41	0.56	0.583
	% Change	97.63 ± 2.47	95.28 ± 2.44	0.82	0.424
	All				
	Learning	37.10 ± 1.38	36.52 ± 1.32	0.60	0.558
	Retrieval	35.95 ± 1.47	35.90 ± 1.57	0.06	0.964
	Change	-1.14 ± 0.81	-0.62 ± 0.59	-0.70	0.493
	% Change	97.18 ± 2.32	98.02 ± 1.70	-0.40	0.692

Numbers indicate either absolute values of correctly recalled pictures after presentation of the word (*Learning* and *Retrieval*), absolute change of correctly recalled associations (*Change*) or change in percentage (*% Change*) and refers to the relative difference from prior to after the retention interval, while the first recall is set to 100%. Therefore a value < 100 % indicates a decrease and a value > 100 % an increase across the retention interval. Differences between cued and uncued associations were tested statistically using paired t-test. Significant differences are marked as bold. Data are means ± SEM

Supplementary Table 2: Arousal ratings for categories 'Emotional' and 'Neutral'

		Cued	Uncued	*t*	*P*
**NREM**	Emotional				
	Baseline	2.49 ± 0.21	2.53 ± 0.21	-0.49	0.629
	Post retention	3.12 ± 0.28	3.18 ± 0.29	-0.87	0.394
	Change	0.63 ± 0.17	0.64 ± 0.21	0.09	0.929
	% Change	128.82 ± 8.70	128.34 ± 9.01	0.05	0.958
	Neutral				
	Baseline	2.37 ± 0.20	2.39 ± 0.19	-0.15	0.882
	Post retention	2.03 ± 0.18	1.94 ± 0.16	0.98	0.337
	Change	-0.34 ± 0.15	-0.44 ± 0.16	-0.87	0.394
	% Change	89.47 ± 6.48	85.82 ± 7.20	0.92	0.367
	All				
	Baseline	2.43 ± 0.20	2.46 ± 0.19	-0.46	0.649
	Post retention	2.57 ± 0.19	2.56 ± 0.18	0.36	0.720
	Change	0.14 ± 0.10	0.10 ± 0.19	0.49	0.629
	% Change	109.71 ± 6.20	105.88 ± 3.83	0.81	0.426
**REM**	Emotional				
	Baseline	2.75 ± 0.23	2.63 ± 0.24	-0.77	0.452
	Post retention	3.65 ± 0.33	2.44 ± 0.26	0.75	0.461
	Change	0.89 ± 0.31	0.76 ± 0.32	-1.12	0.278
	% Change	151.35 ± 22.83	150.05 ± 23.89	0.28	0.781
	Neutral				
	Baseline	2.62 ± 0.26	2.69 ± 0.25	-0.65	0.562
	Post retention	2.44 ± 0.26	2.24 ± 0.24	1.54	0.141
	Change	-0.18 ± 0.32	-0.45 ± 0.15	-1.56	0.136
	% Change	96.55 ± 6.75	85.38 ± 4.86	1.85	0.080
	All				
	Baseline	2.70 ± 0.23	2.75 ± 0.24	-0.88	0.391
	Post retention	3.05 ± 0.23	2.91 ± 0.23	1.61	0.125
	Change	0.35 ± 0.17	0.15 ± 0.18	1.69	0.108
	% Change	124.58 ± 14.07	116.42 ± 12.76	1.85	0.079
**Wake**	Emotional				
	Baseline	2.52 ± 0.20	2.35 ± 0.17	1.15	0.262
	Post retention	3.30 ± 0.33	3.40 ± 0.33	-1.06	0.302
	Change	0.78 ± 0.31	1.05 ± 0.28	1.71	0.103
	% Change	142.50 ± 17.32	148.55 ± 16.50	0.55	0.589
	Neutral				
	Baseline	2.33 ± 0.24	2.50 ± 0.24	-1.48	0.155
	Post retention	1.85 ± 0.24	2.08 ± 0.20	-2.73	**0.013**
	Change	-0.48 ± 0.15	-0.42 ± 0.17	0.41	0.690
	% Change	82.89 ± 6.15	87.33 ± 6.67	-0.68	0.502
	All				
	Baseline	2.42 ± 0.21	2.43 ± 0.19	-0.05	0.961
	Post retention	2.57 ± 0.20	2.74 ± 0.23	-2.29	0.033
	Change	0.15 ± 0.14	0.31 ± 0.16	-1.60	0.126
	% Change	111.06 ± 8.69	115.28 ± 7.97	-0.95	0.355

Numbers indicate absolute arousal ratings for emotional or neutral pictures after presentation of the word (*Learning* and *Retrieval*), either before the association learning task (baseline) or after learning and the retention interval. Differences between the two ratings are indicated as absolute change (*Change*) or change in percentage *(% Change*), referring to the relative difference from baseline to after the retention interval, while the first rating is set to 100%. Therefore, a value < 100 % indicates a decrease and a value > 100 % an increase across the retention interval. Differences between cued and uncued associations were tested statistically using paired t-test. Significant differences are marked as bold. Data are means ± SEM

Supplementary Table 3: Expectancy ratings for categories 'Emotional' and 'Neutral

		Cued	Uncued	*t*	*P*
**NREM**	Emotional				
	Learning	3.23 ± 0.08	3.19 ± 0.08	1.58	0.211
	Post retention	3.15 ± 0.10	3.06 ± 0.10	1.78	0.092
	Change	-0.08 ± 0.04	-0.13 ± 0.04	1.13	0.274
	% Change	97.71 ± .1.24	95.53 ± 1.39	1.15	0.266
	Neutral				
	Learning	3.34 ± 0.08	3.26 ± 0.08	1.32	0.341
	Post retention	3.23 ± 0.09	3.18 ± 0.04	1.04	0.317
	Change	-0.10 ± 0.04	-0.08 ± 0.05	-0.23	0.824
	% Change	96.84 ± 1.34	97.35 ± 1.57	-0.28	0.782
	All				
	Learning	3.28 ± 0.07	3.23 ± 0.07	1.21	0.110
	Post retention	3.20 ± 0.08	3.11 ± 0.08	2.19	**0.045**
	Change	-0.08 ± 0.03	-0.12 ± 0.04	0.67	0.51
	% Change	97.24 ± 0.87	96.45 ± 1.17	0.65	0.52
**REM**	Emotional				
	Learning	3.03 ± 0.12	2.99 ± 0.11	1.70	0.141
	Post retention	2.98 ± 0.12	2.96 ± 0.12	0.38	0.791
	Change	-0.05 ± 0.06	-0.03 ± 0.04	-0.40	0.710
	% Change	98.57 ± 2.05	98.96 ± 1.76	-0.20	0.842
	Neutral				
	Learning	3.19 ± 0.09	3.16 ± 0.10	1.25	0.223
	Post retention	3.11 ± 0.10	3.12 ± 0.11	-0.07	0.925
	Change	-0.08 ± 0.05	-0.04 ± 0.06	-0.71	0.491
	% Change	97.48 ± 1.70	98.78 ± 1.99	-0.68	0.560
	All				
	Learning	3.11 ± 0.09	3.07 ± 0.09	1.50	0.154
	Post retention	3.05 ± 0.09	3.04 ± 0.10	0.19	0.853
	Change	-0.06 ± 0.03	-0.03 ± 0.04	-0.76	0.426
	% Change	97.87 ± 1.20	98.81 ± 1.39	-0.67	0.571
**Wake**	Emotional				
	Learning	2.92 ± 0.12	2.87 ± 0.12	1.23	0.281
	Post retention	2.85 ± 0.12	2.90 ± 0.12	-0.98	0.364
	Change	-0.07 ± 0.05	0.03 ± 0.04	-2.03	0.056
	% Change	97.71 ± 1.75	101.36 ± 1.46	-2.14	0.085
	Neutral				
	Learning	3.24 ± 0.11	3.16 ± 1.10	1.08	0.352
	Post retention	3.11 ± 0.09	2.98 ± 0.11	1.57	0.133
	Change	-0.13 ± 0.06	-0.18 ± 0.06	0.56	0.548
	% Change	96.71 ± 1.75	94.58 ± 1.82	0.81	0.434
	All				
	Learning	3.08 ± 0.09	3.01 ± 0.09	1.87	0.225
	Post retention	2.98 ± 0.09	2.94 ± 0.09	0.76	0.436
	Change	-0.10 ± 0.04	-0.07 ± 0.04	-0.58	0.578
	% Change	97.06 ± 1.44	97.76 ± 1.26	-0.47	0.684

Numbers indicate absolute expectancy ratings for words either before the association learning task (*Baseline*) or after learning and the retention interval (*Post retention*). Differences between the two ratings are indicated as absolute change (*Change*) or change in percentage *(% Change*), referring to the relative difference from baseline to after the retention interval, while the first rating is set to 100%. Therefore a value < 100 % indicates a decrease and a value > 100 % an increase across the retention interval. Differences between cued and uncued associations were tested statistically using paired t-test. Significant differences are marked as bold. Data are means ± SEM

Baseline performance and arousal ratings

After learning before the retention interval, participants generally recalled significantly more emotional associations (73.33 ± 1.66 %) as compared to neutral associations (70.19 ± 1.78 %, t_{61} = 2.43, P = 0.018, η_p^2 = 0.05), indicating the well-known modulating influence of emotion during encoding on memory performance. Importantly, no baseline differences in learning performance were observed between the NREM, REM and wake groups and cued and uncued word-picture associations, and no interactions with emotional arousal occurred (all P > 0.20, see **Supplementary Table 1** for details). Thus, we can safely exclude baseline differences in our study. Interestingly, within the emotional category, emotionally high arousing (73.41 ± 1.85 %) were better remembered as compared to emotionally low arousing associations (71.34 ± 1.93 %; t_{61} = 2.21, P = 0.038, η_p^2 = 0.06). Before the emotional association task, neutral words were rated as emotionally neutral, and these arousal ratings did not differ between words later associated with either high or low arousing pictures and were either cued or not (P > 0.40). This was true for all three groups individually (see **Supplementary Table 2**, for descriptive values). After the whole procedure (including the associative emotional memory task, the retention interval and the delayed cued recall testing), words associated with emotional pictures were rated as more arousing as compared to words paired with low arousing pictures (139.80± 9.32 % vs. 87.14 ± 3.27%; t_{61} = 5.53, P < 0.001, η_p^2 = 0.43; with arousal rating at baseline set to 100%; see **Supplementary Table 2**). Reflecting the emotional enhancement effect on baseline cued recall performance, words associated with emotionally high arousing (155.22± 12.02 %) were perceived significantly more arousing as compared to words associated with emotionally low arousing picture (130.04± 7.93 %; t_{61} = 3.13, P = 0.003, η_p^2 = 0.05). Thus, the arousal of the pictures generalized to arousal ratings of the word alone (without picture presentation). However, changes in arousal ratings did not significantly differ between the three groups nor did we observe an interaction with cueing of associations (all P > 0.19, **Supplementary Fig. 1**, see **Supplementary Table 2** for absolute values).

Oscillatory correlates of successful memory cueing during REM sleep

One might argue that the topographic distribution of power increases associated with subsequent cueing effects might be different during NREM and REM sleep, and that the use of the ROI obtained from NREM sleep might be not appropriate here. Thus, we analyzed the oscillatory correlates of successful memory cueing also during REM sleep alone. Consistent with our findings reported above, we observed no significant cluster of electrodes during REM sleep that exhibited increased power in the spindle range for later remembered as compared to later forgotten cued associations. For theta power, we again observed a significant cluster of electrodes with increased power for later remembered vs. later forgotten trials (P = 0.024, corrected for multiple comparisons, cluster see **Fig. 3K**). The cluster contained fewer electrodes as the cluster observed during NREM sleep, which consisted in fact of a subset of electrodes of those significantly activated by cueing during NREM sleep. Similar to the results reported above, the increase in theta activity was mainly seen for later remembered neutral associations (t_{16} = 3.74, P = 0.002, η_p^2 = 0.28), whereas theta activity in emotional and later remembered cueing did not differ significantly from forgotten cueing trials (t_{16} = 1.77, P = 0.094, η_p^2 = 004). Furthermore, the two categories of remembered associations differed significantly (t_{16} = -2.28, P = 0.037, η_p^2 = 0.08).
